# On the Analysis of PM/FM Noise Radar Waveforms Considering Modulating Signals with Varied Stochastic Properties

**DOI:** 10.3390/s21051727

**Published:** 2021-03-03

**Authors:** Leandro Pralon, Gabriel Beltrao, Alisson Barreto, Bruno Cosenza

**Affiliations:** 1 Brazilian Army Technological Center, 23020-470 Rio de Janeiro, Brazil; barreto.alisson@eb.mil.br (A.B.); bruno.cosenza@eb.mil.br (B.C.); 2 SnT, University of Luxembourg, L-1855 Luxembourg, Luxembourg; gabriel.tedgue-beltrao@uni.lu

**Keywords:** noise radar, random waveform, phase/frequency modulation, ambiguity function, real-time implementation, randomness

## Abstract

Noise Radar technology is the general term used to describe radar systems that employ realizations of a given stochastic process as transmit waveforms. Originally, carriers modulated in amplitude by a Gaussian random signal, derived from a hardware noise source, were taken into consideration, justifying the adopted nomenclature. With the advances made in hardware as well as the rise of the software defined noise radar concept, waveform design emerges as an important research area related to such systems. The possibility of generating signals with varied stochastic properties increased the potential in achieving systems with enhanced performances. The characterization of random phase and frequency modulated waveforms (more suitable for several applications) has then gained considerable notoriety within the radar community as well. Several optimization algorithms have been proposed in order to conveniently shape both the autocorrelation function of the random samples that comprise the transmit signal, as well as their power spectrum density. Nevertheless, little attention has been driven to properly characterize the stochastic properties of those signals through closed form expressions, jeopardizing the effectiveness of the aforementioned algorithms as well as their reproducibility. Within this context, this paper investigates the performance of several random phase and frequency modulated waveforms, varying the stochastic properties of their modulating signals.

## 1. Introduction

Noise Radars are electromagnetic systems that use random signals to detect and locate reflecting objects [1]. Even though such concept had been defined in the early post WWII days [2,3], random transmit waveforms were left aside for a long time due to their high generation complexity, often requiring a controlled hardware noise source. The advances made in hardware enabled the generation of stochastic processes with varied probabilistic properties. Therefore, signals of that nature started gaining attention, giving rise to the software defined noise radar concept. As a consequence, the investigation of the most suitable stochastic properties of the transmit waveforms, as well as the proper characterization of the signals throughout the receiver’s processing chain emerged as important research areas related to such systems.

The stochastic nature of the transmit waveforms make them intuitively attractive for applications that require high performance against external interferences (intentional or not) and low probability of interception (LPI). In addition, if system parameters are properly chosen, several other features can be achieved as well. This is the case of high electromagnetic compatibility, with low cross-correlation properties between pulses transmitted at different time. The latter can lead both to the suppression of range ambiguity as well as an efficient spectrum sharing with other systems. Good range and Doppler resolution, low range side lobes, and short blind zones (in pulsed radars) can also be achieved if properly taken into consideration during the waveform and/or receiver processing chain design procedures.

With respect to the receiver processing chain design, matched filtering followed by pulse integration is the most traditional configuration employed in Noise Radar systems. Nevertheless, mismatched filtering [4], apodization filtering [5], and other types of post processing optimization algorithms like CLEAN [6,7] have also been proposed, especially to minimize the side lobes levels at the pulse compression outputs. Aside from the higher computational cost inherent to the aforementioned algorithms, they may also introduce undesired effects, like a decrease in the signal to noise ratio or a range resolution deterioration.

Waveform generation, in turn, is one of the most active research areas within the Noise Radar community, and many different approaches have already been proposed, e.g., [8,9,10,11,12,13]. Being relatively easy to model and generate, carriers modulated in amplitude by a random signal are the most popular ones. Generally, noise sources that produce signals with Gaussian amplitude and flat power spectral density are employed, justifying the adopted nomenclature, “Noise Radar” [9,10,14]. Aside from having an elevated degree of randomness, Gaussian processes’ properties are easy to work with and perform well in a pulse compression radar architecture.

Nevertheless, phase and frequency modulated (PM/FM) signals suit better systems that require high power efficiency, since the employment of constant envelope signals result in a lower peak-to-average power ratio. Therefore, as an alternative to amplitude modulation, great effort has been spent in deriving closed form expressions of pseudo-random PM/FM signals that better fulfill radar systems requirements. As examples, in [15,16], a hybrid approach is proposed, combining linear frequency modulation with a random phase scaling factor, while in [17], the authors propose a waveform whose modulation function is represented by a Fourier series expansion with coefficients generated using independent identically distributed (i.i.d) Gaussian random variables. Several optimization algorithms have also been proposed to generate constant modulus pseudo-random waveforms with specific desired features, either by optimizing the Radar Ambiguity Function [18,19] or properly shaping its spectral response [20,21,22].

A few drawbacks may arise with the employment of such waveforms. First, the authors in [23] brought up the question about the degree of randomness and how modified pseudo-random waveforms perform in electronic warfare environments. Since they all significantly differ from realizations of a white Gaussian stochastic process, their LPI intrinsic property may be considerably compromised. The cross correlation function of such waveforms may also change during the optimization procedure, a subject seldom addressed in the literature, but that may have significant impact in several applications. Finally, the performance of the optimized waveforms, especially with respect to sidelobes, may also be compromised by the target’s Doppler shift [24], limiting their employment. In summary, when optimizing, no closed form expressions of the generated waveforms are provided. Therefore, their correct characterization as well as their generation in real time applications becomes difficult, jeopardizing the reproducibility of the results.

The work initiated by Axelsson [12], on the other hand, focused on deriving first and second order moment closed form expressions describing the signals throughout a PM/FM Noise Radar receiver processing chain, composed by matched filtering and coherent integration. One of the main contributions of the aforementioned work was the derivation of closed form expressions that describe the expected value of the matched filter output power. The latter enabled a proper analytical analysis of, among other parameters, the system’s range resolution and its stationary side lobes level. Nevertheless, in the referred work, little attention was given to the particularities introduced by the stochastic properties of the modulating signal. In [8] this study was extended, considering a special type of Gaussian modulating signal with flat power spectral density. Considerations on its bandwidth and variance were made, leading to simpler and easier expressions to work with, but still without properly addressing the stochastic nature of circular distributions that characterize the phase of constant modulus random vectors, which are defined around the unit circle.

Within this context, this paper focuses on the analysis of phase and frequency modulated waveforms, considering mainly, but not limited to Wrapped Normal modulating signals with varied stochastic properties, that are, at most, windowed. It is important to highlight that no optimization procedure is considered in the study. The comparison is made by a detailed analysis of the closed form expressions characterizing the first and second order moments of the ambiguity function. Formulations describing the main lobe, the side lobes level at infinity (ISL), and the random peak side lobe (PSL) at the matched filter output are then analyzed with different parameters, always considering a possible Doppler shift introduced by moving targets. Unlike ISL, which can be estimated under a stationary assumption, the analysis of PSL is not straightforward since, depending on the modulating signal and on the modulation procedure, it will most likely occur in a high variance transient region between a nearly zero variance region (main lobe) and a stationary variance region (ISL).

Bench top experiments are also performed to evaluate the performance of such waveforms in band limited systems. Despite presenting high spectral efficiency with respect to the spectrum sharing point of view, the side lobes level at the matched filter output are highly dependent on the Power Spectral Density of the transmit signal. Since PM/FM random waveforms do not present a perfect spectral containment, both the ISL as well as the PSL at the matched filter output can be deteriorated if filtering operations, either in hardware or in software (as in digital down converters), are not properly executed. A discussion about the degree of randomness of PM/FM pseudorandom waveforms is also presented, in an attempt to access their performance in electronic warfare environments. Finally, the feasibility of generating such transmit signals in real time applications is addressed, considering a simple FPGA implementation based on Linear Feedback Shift Registers.

The remainder of this paper is organized as follows. In Section 2, a discussion of PM/FM Noise Radar transmit signal’s ambiguity function is addressed, in light of Directional Statistics principles. In Section 3, Wrapped Normal modulating signals with different stochastic properties are presented as well as the corresponding expected matched filter output powers. Simulation and bench top experiments results in accordance with the analytical analysis performed are also presented. Section 3 also addresses the design and details relative to the RTL synthesis and FPGA implementation of a few of the analyzed waveforms. Finally, in Section 4 conclusions are drawn.

## 2. PM/FM Noise Radar and Directional Statistics

In the present section, the basic principles of PM/FM Noise Radar systems are addressed, introducing relevant concepts related to Directional Statistics used in the initial formulation of the problem. In Noise Radar systems, the transmit signal is characterized by a stochastic process, s(t), i.e., different sample functions of s(t) are transmitted in distinct intervals. As a consequence, the corresponding matched filter outputs, relative to pulse compression architectures, are all characterized by complex random processes. It is then mandatory that the proper analysis of Noise Radars involves the use of probabilistic tools, as is the case with the derivation of a random signal’s ambiguity function, that describes the matched filter output as a function of a presumable Doppler shift caused by the target radial velocity [25]. It’s expected value is given by

(1) $$\begin{array}{c} E\left[\chi_{\tilde{s}}\left(\tau ,f_{d}\right)\right]=E\left[\int_{-\infty }^{\infty }\tilde{s}\left(t\right){\tilde{s}}^{*}\left(t-\tau \right)e^{j2\pi f_{d}t}dt\right] \end{array}$$

where $\tilde{s}\left(t\right)$ is the complex envelope of s(t), $f_{d}$ is the Doppler frequency, τ is the time delay and *j* is the imaginary unity.

In pulsed radars, the transmit signal is time limited with duration $\tau_{s}$, characteristic that is mathematically represented by the rectangular function ${rect}_{\tau_{s}}\left(t\right)$, in order to assure that the process $\tilde{s}\left(t\right)$ remains stationary. Furthermore, assuming that $f_{d}$ is deterministic, the expectation in (1) is over the distribution of $\tilde{s}\left(t\right)$, therefore, (1) can be rewritten as

(2) $$\begin{array}{c} E\left[\chi_{\tilde{s}}\left(\tau ,f_{d}\right)\right]=\int_{-\infty }^{\infty }E\left[\tilde{s}\left(t\right){\tilde{s}}^{*}\left(t-\tau \right)\right]{rect}_{\tau_{s}}\left(t\right)e^{j2\pi f_{d}t}dt, \end{array}$$

Assuming $\tilde{s}\left(t\right)$ to be stationary, (2) can be further simplified as

(3) $$\begin{array}{c} E\left[\chi_{\tilde{s}}\left(\tau ,f_{d}\right)\right]=\tau_{s}R_{\tilde{s}}\left(\tau \right)sinc\left(f_{d}\tau_{s}\right), \end{array}$$

where $R_{\tilde{s}}\left(\tau \right)=E\left[\tilde{s}\left(t\right){\tilde{s}}^{*}\left(t-\tau \right)\right]$ is the autocorrelation function of the complex stochastic process $\tilde{s}\left(t\right)$ [26]. Despite being relatively intuitive, the near thumbtack format depicted in (3) shows that that the expected range and Doppler profiles in Noise Radar systems are independent functions; therefore, no range-Doppler coupling is expected in Noise Radars [27].

Let us now consider that the transmitted signals are time limited carriers, modulated in phase or frequency, with complex envelopes, with respect to the central transmit frequency, $\tilde{s}\left(t\right)=\sqrt{2P}\;e^{j[\theta (t)+\phi ]}\;\;\;,\;\;\;0<t<\tau_{s}$, where 2P is the signal’s complex envelope mean power, ϕ is a random phase, assumed to be uniformly distributed in (0,2π], and θ(t) represents the carrier modulated phase, the latter being a function of the stochastic process characterizing the modulating signal a(t), written as

(4) $$\begin{array}{c} \theta \left(t\right)=\left\{\begin{array}{cc} a(t) & ,for\;phase\;modulation\;(PM);\\ \int_{-\infty }^{t}a\left(\alpha \right)d\alpha & ,for\;frequency\;modulation\;(FM). \end{array}\right. \end{array}$$

In classical radars (that employ deterministic signals), waveform design involves selecting an appropriate type of biphase/polyphase code, under the PM assumption, or the most suitable frequency function of time, either linear or nonlinear (generally based on the principal of stationary phase [28]), when FM is employed. Analogously, in Noise Radars, properly choosing the stochastic properties of the modulating signal will directly affect the system’s overall performance. For example, the PM/FM transmit signal’s autocorrelation function in (3) is defined as

(5) $$\begin{array}{c} R_{\tilde{s}}\left(t_{1},t_{2}\right)=2PE\left[e^{j(\theta \left(t_{1}\right)-\theta \left(t_{2}\right))}\right] \end{array}$$

Note from (3) and (5) that the first mandatory stochastic property that the transmit signal complex envelope, $\tilde{s}\left(t\right)$, has to present is that it has to be wide sense stationary, implying that its autocorrelation function depends on the difference $\tau =t_{1}-t_{2}$. A sufficient condition to meet the later is that the process θ(t) in (4) is 2nd order stationary [26], which is assumed to be true throughout the derivation.

Let us now consider the random variable $\psi_{\tau }=\theta \left(t_{1}\right)-\theta \left(t_{2}\right)$. If $\psi_{\tau }$ has a probability density function $p(\psi_{\tau })$, its characteristic function is given by the Fourier transform of the latter, i.e.,

(6) $$\begin{array}{c} M_{\psi_{\tau }}\left(v\right)=E\left[e^{jv\psi_{\tau }}\right]=\int_{R}e^{jv\psi_{\tau }}p(\psi_{\tau })d\psi_{\tau } \end{array}$$

The autocorrelation function of the transmit signal in (5) can then be rewritten as

(7) $$\begin{array}{c} R_{\tilde{s}}\left(\tau \right)=2PM_{\psi_{\tau }}\left(1\right) \end{array}$$

Considering (3) and (7), it becomes evident the substantial role of the modulating signal in Noise Radar systems, especially in obtaining transmit signals with narrow and bell-shaped autocorrelation functions, with low side lobe levels. Furthermore, according to the Wiener–Kintchine theorem, the transmit signal’s power spectral density, $S_{\tilde{s}}\left(f\right)$, is given by the Fourier transform of $R_{\tilde{s}}\left(\tau \right)$. Therefore, the modulating signal has also direct influence on the system spectral efficiency/containment, a parameter whose importance is rapidly increasing due to the scarcity of the RF spectrum and the harder constraints on coexisting systems in the context of multi application spectrum sharing [28]. Within this context, (7) can be considered the basis of PM/FM Noise Radar waveform design. Nevertheless, little attention has been given in exploiting both its constraints as well as its features under the assumption of varied stochastic properties of the modulating signals.

First it is worth mentioning that ψ(τ), being the phase of a random vector is characterized by a continuous (with respect to the Lebesgue measure on a circumference) random variable with a circular distribution, i.e., its total probability is concentrated on the circumference of a unit circle [29,30,31]. Its probability density function $p(\psi_{\tau })$ is positive, and periodic, i.e., $p(\psi_{\tau })=p(\psi_{\tau }+2k\pi )$, for any k∈Z and when *v* in (6) equals 1, it reduces to [29]

(8) $$\begin{array}{c} M_{\psi_{\tau }}\left(1\right)=\rho e^{jm} \end{array}$$

where ρ is a function of the concentration of the $\psi_{\tau }$ angle and *m* its mean direction. When $\psi_{\tau }$ is characterized by an uniform distribution in (0,2π], for example, ρ=0, and therefore this distribution has no well defined mean, being referred to as simply isotropic.

Defining stochastic processes θ(t) that result in distributions to characterize $\psi_{\tau }$ that better fulfill radar systems requirements, including their feasibility of generation, is not a simple task. Furthermore, depending on the chosen distribution, standard statistical methods and rules, defined for data in the Euclidean space, may not apply as expected, leading to incorrect modeling.

The research area related to Directional Statistic is very active, e.g., [29,30,31], just to cite a few. One of the most popular models for directional data is the Von Mises [30] distribution, whose probability density function for a circular random variable θ∈[0,2π) is given by [29]

(9) $$\begin{array}{c} p\left(\theta \right)=\frac{1}{2\pi I_{0}\left(k\right)}e^{k\cos(\theta -m)} \end{array}$$

where $I_{0}\left(k\right)=\sum_{r=0}^{\infty }{\left(k/2\right)}^{2r}{\left(1/r!\right)}^{2}$ is the modified Bessel function of the first kind and order zero, 0≤m≤2π is the mean direction and 0≤k measures the concentration around *m*.

Due to its similarities to the Normal distribution on the real line and importance in modeling several circular data in most real life problems, the Von Mises distribution is also often called Circular Normal. It is symmetric about *m*, unimodal (presents a preferred direction), it presents a maximum likelihood estimator of *m* and is the circular distribution with maximum entropy. When ψ(τ) is characterized by a Von Mises distribution, (8) is written by

(10) $$\begin{array}{c} M_{\psi_{\tau }}\left(1\right)=\frac{I_{1}\left(k\right)}{I_{0}\left(k\right)}e^{jm}\;\;\;\;,\mathrm{with}\;\;\;\;I_{1}\left(k\right)=\frac{k}{2}\sum_{r=0}^{\infty }\frac{{\left(k^{2}/4\right)}^{r}}{r!\;\Gamma (r+2)} \end{array}$$

where Γ(z) is the gamma function.

Furthermore, for large values of *k*, (9) approaches the linear Normal distribution with zero mean and unit variance [29]. Despite the aforementioned features, in Noise Radar applications it is desired that the random process characterizing the modulating signal of the transmit waveforms is easy to generate and retains the additive property, related to the convolution, of two random variables belonging to the same distribution, which the Von Mises distribution do not present. The Wrapped Normal distribution, on the other hand, can be obtained by wrapping a linear Normal distribution around 2π, having a pdf defined by [29]

(11) $$\begin{array}{c} p\left(\theta \right)=\frac{1}{\sigma \sqrt{2\pi }}\sum_{m=-\infty }^{\infty }exp\left[\frac{-{\left(\theta -m-2\pi m\right)}^{2}}{2\sigma^{2}}\right] \end{array}$$

where *m* and σ are the mean and standard deviation of the linear Normal distribution.

Evaluation of (11) is not trivial, since it involves an infinite series; nevertheless, the Wrapped Normal distribution is not only easy to generate but it is also associated to the central limit theorem of circular random variables and is closed under convolution (presents the additive property), i.e., if $\theta_{1}∼WN\left(m_{1},\sigma_{1}\right)$ and $\theta_{2}∼WN\left(m_{2},\sigma_{2}\right)$, then $\theta_{1}+\theta_{2}∼WN\left(m_{1}+m_{2},\sigma_{1}\sigma_{2}\right)$.

As stated in [32], the similarities between the Wrapped Normal distributions and Circular Normal distributions can be used in a complementary fashion, since there are statistical contexts where each one can be more convenient than the other. We believe that this is the case for FM/PM Noise Radar applications.

## 3. Wrapped Normal Modulating Signals

As discussed in the previous section, the Wrapped Normal (WN) distribution poses as a good model to characterize random variables from the stochastic process that defines an FM/PM Noise Radar modulating signal. The Wrapped Normal distribution is a particular case of the Symmetric Wrapped α-Stable family of circular densities, that share all the previously mentioned properties of WN distributions. The probability density function of such family, as a function of the parameter α is given by

(12) $$\begin{array}{c} p\left(\theta \right)=\frac{1}{2\pi }+\frac{1}{\pi }\sum_{k=1}^{\infty }exp\left(\sigma^{\alpha }\right)\cos\left[k\left(\theta -m\right)\right] \end{array}$$

Symmetric Wrapped Stable distributions also present an important property related to their characteristic functions since, for any integer value, they correspond to the characteristic function of their unwrapped versions [29]. Therefore, and considering their above mentioned additive property, without loss of generality, (7) can be derived assuming a(t) to be linear, Gaussian and wide sense stationary [33], and, consequently, θ(t) in (4) to be also linear, Gaussian, and wide sense stationary. The latter holds both when phase modulation is assumed as well as when frequency modulation is taken into consideration, since the integrator is a linear time invariant filter.

Noting that Gaussian processes that are wide sense stationary are also strict sense stationary and, therefore, are 2nd order stationary, it is guaranteed that the complex envelope $\tilde{s}\left(t\right)$ is wide sense stationary. Furthermore, when the time limited signal, $\tilde{s}\left(t\right)$, is modulated in phase or frequency by a Gaussian wide sense stationary random process,

(13) $$\begin{array}{c} M_{\psi_{\tau }}\left(1\right)=e^{jm}e^{\frac{-\sigma^{2}}{2}} \end{array}$$

where *m* and $\sigma^{2}$ are the mean and variance of $\psi_{\tau }$, respectively. Hence, the transmit signal’s autocorrelation function reduces to [12]

(14) $$\begin{array}{c} R_{\tilde{s}}\left(\tau \right)=2Pe^{-[R_{\theta }\left(0\right)-R_{\theta }\left(\tau \right)]} \end{array}$$

where $R_{\theta }\left(\tau \right)$ is the autocorrelation of the θ(t) angle.

The ambiguity function first order moment in (3) is a good indicator of the waveform’s performance. Nevertheless, to properly analyze both the system’s range resolution as well as its side lobe levels, it is necessary to investigate the second order moment of the stochastic process that describe the signal at the matched filter output. Let us then investigate the second order moment of the ambiguity function of Noise Radars transmit waveforms, given by [26]

(15) $$\begin{array}{c} E\left[|\chi_{\tilde{s}}\left(\tau ,f_{d}\right){|}^{2}\right]=E\left[\chi_{\tilde{s}}\left(\tau ,f_{d}\right)\chi_{\tilde{s}}^{*}\left(\tau ,f_{d}\right)\right] \end{array}$$

Expanding (15) yields

(16) $$\begin{array}{c} E\left[|\chi_{\tilde{s}}\left(\tau ,f_{d}\right){|}^{2}\right]\;\;=\;\;\;\;\int_{-\infty }^{\infty }\int_{-\infty }^{\infty }\;\;E\left[{\tilde{s}}_{i}\left(\beta +\tau \right)\;\;{\tilde{s}}_{i}^{*}\left(\beta \right)\;\;e^{j2\pi f_{d}\beta }{rect}_{\tau_{s}}\left(\beta \right)\;\cdot \;\;{\tilde{s}}_{i}^{*}\left(\alpha +\tau \right)\;\;{\tilde{s}}_{i}\left(\alpha \right)\;\;e^{-j2\pi f_{d}\alpha }{rect}_{\tau_{s}}\left(\alpha \right)\right]\;\;d\alpha \;\;d\beta \end{array}$$

Assuming that the complex envelope ${\tilde{s}}_{i}\left(t\right)$ is phase or frequency modulated, it is possible to write

(17) $$\begin{array}{c} E\left[|\chi_{\tilde{s}}\left(\tau ,f_{d}\right){|}^{2}\right]=\;\;\;\;\int_{-\infty }^{\infty }\int_{-\infty }^{\infty }E\left[e^{jz(\alpha ,\beta )}\right]\;e^{j2\pi f_{d}\beta }{rect}_{\tau_{s}}\left(\beta \right)e^{-j2\pi f_{d}\alpha }{rect}_{\tau_{s}}\left(\alpha \right)]\;d\alpha \;d\beta \end{array}$$

with z(α,β) being the random variable defined as

(18) $$\begin{array}{c} z(\alpha ,\beta )\;=\;\theta (\beta \;+\;\tau )\;-\;\theta (\beta )\;-\;\theta (\alpha \;+\;\tau \;)+\;\theta (\alpha ) \end{array}$$

Since θ(t) is a zero mean Gaussian random process, the random variable z(α,β) is also Gaussian with zero mean. At this point it is important to remember that, as previously discussed, throughout the present work, the linear Normal model is being used instead of the Wrapped Normal one, with no loss of generality. After some mathematical manipulations, analogous to what has been done for the zero Doppler particular case in [8], it is possible to obtain

(19) $$\begin{array}{ccc} \;\;\;\;\;\;\;\; & & \;\;\;\;\;\;\;\;E\left[|\chi_{\tilde{s}}\left(\tau ,f_{d}\right){|}^{2}\right]=4P^{2}\int_{-\infty }^{\infty }\int_{-\infty }^{\infty }\;\;\;\;\exp\left\{-\;\;2R_{\theta }\left(0\right)+2R_{\theta }\left(\tau \right)+\right.\\ & & \left.+\;2R_{\theta }\left(\alpha -\beta \right)-R_{\theta }\left(\alpha -\beta \;+\;\tau \right)-R_{\theta }\left(\alpha -\beta \;-\;\;\tau \right)\right\}{rect}_{\tau_{s}}\left(\alpha \right){rect}_{\tau_{s}}\left(\beta \right)e^{-j2\pi f_{d}\left(\alpha -\beta \right)}d\alpha d\beta \end{array}$$

Noting that the integrator in (19) is a function of the difference γ=α−β and that $R_{\theta }\left(x\right)$ is an even function of *x*, it is possible to reduce the double integral in (19) to a single integral, obtaining

(20) $$\begin{array}{ccc} E\left[|\chi_{\tilde{s}}\left(\tau ,f_{d}\right){|}^{2}\right]\;\;\;\; & = & \;\;\;\;4P^{2}\tau_{s}\int_{-\infty }^{\infty }tri\left(\gamma /\tau_{s}\right)\cdot \\ & & \;\;\cdot \exp\left\{-\;\;2R_{\theta }\left(0\right)+2R_{\theta }\left(\tau \right)+\;2R_{\theta }\left(\gamma \right)-R_{\theta }\left(\gamma \;+\;\tau \right)-R_{\theta }\left(\gamma \;-\;\;\tau \right)\right\}e^{-j2\pi f_{d}\gamma }d\gamma \end{array}$$

where $tri(\gamma /\tau_{s})$ is the triangular function with duration $2\tau_{s}$, given by

(21) $$\begin{array}{c} tri\left(\gamma /\tau_{s}\right)=\left\{\begin{array}{ccc} 1-|\gamma /\tau_{s}| & ; & \left|\gamma \right|\leq \tau_{s}\\ 0 & ; & |\gamma |>\tau_{s}. \end{array}\right. \end{array}$$

Analogous to previous analysis [8,12], two regions can be identified in (20), that provide good approximations for both the main lobe as well as the stationary side lobe level. First, note that, if τ is close to 0, except for values of γ close to zero, $2R_{\theta }\left(\gamma \right)\approx R_{\theta }\left(\gamma \;+\;\tau \right)+R_{\theta }\left(\gamma \;-\;\;\tau \right)$, which leads to

(22) $$\begin{array}{c} E{\left[|\chi_{\tilde{s}}\left(\tau ,f_{d}\right){|}^{2}\right]}_{ML}=4P^{2}\tau_{s}\int_{-\infty }^{\infty }tri\left(\gamma /\tau_{s}\right)\exp\left\{-\;\;2R_{\theta }\left(0\right)+2R_{\theta }\left(\tau \right)\right\}e^{-j2\pi f_{d}\gamma }d\gamma \end{array}$$

The integral in (22) becomes the Fourier transform of the aforementioned triangle function and hence

(23) $$\begin{array}{c} E{\left[|\chi_{\tilde{s}}\left(\tau ,f_{d}\right){|}^{2}\right]}_{ML}={\tau_{s}}^{2}R_{s}^{2}\left(\tau \right){sinc}^{2}\left(f_{d}\tau_{s}\right) \end{array}$$

We refer to this result as the main lobe characterization. The importance of this result is unquestionable since target detection is directly obtained from the maximum of this function as well as the range and Doppler resolution from the 3 dB widths in both dimensions. Note the small Doppler tolerance of such type of waveforms, which is not suitable for search radars (when matched filtering is employed).

Now, let us address the behavior of what can be considered the side lobes levels characterization, i.e., the behavior of what can be considered an upper bound to the side lobes levels [34]. For large values of τ, (20) presents a stationary nature and, after some mathematical manipulations, it can be shown that the expected value of the stationary side lobe level power (SSL) or, the equivalent side lobe level in the infinity (ISL), is independent of τ and is given by

(24) $$\begin{array}{c} E{\left[|\chi_{\tilde{s}}\left(\tau ,f_{d}\right){|}^{2}\right]}_{ISL}=4P^{2}\tau_{s}\int_{-\infty }^{\infty }tri\left(\gamma /\tau_{s}\right)\exp\left\{-\;\;2R_{\theta }\left(0\right)+\;2R_{\theta }\left(\gamma \right)\right\}e^{-j2\pi f_{d}\gamma }d\gamma \end{array}$$

Simplifying, it is possible to write

(25) $$\begin{array}{c} E{\left[|\chi_{\tilde{s}}\left(\tau ,f_{d}\right){|}^{2}\right]}_{ISL}=\tau_{s}\int_{-\infty }^{\infty }tri\left(\gamma /\tau_{s}\right)R_{s}^{2}\left(\gamma \right)e^{-j2\pi f_{d}\gamma }d\gamma \end{array}$$

Equation (25) dictates the noise floor, or random fluctuation level, that arises from the stochastic side lobes of the matched filter output. Many works have been proposed in the literature to either reduce or minimize the referred fluctuations, since they can completely mask other targets, e.g., [35,36,37]. Filter design, both during the generation of the transmit waveforms [13] as well as combined with the pulse compression operation (mismatched filter [4]), have also been subject of many studies, generally based on recursive optimization procedures. Nevertheless, such type of algorithms not only insert an amount of complexity that some systems may not cope with but also change the stochastic properties of the transmit signals. Therefore, in order to better propose optimization algorithms it is first necessary to correctly describe the mathematical formulation of the referred noise floor as a function of the transmit signal’s parameters.

The rule of thumb, widely spread within the Noise Radar community, is that the peak to stationary side lobe level (or side lobe at infinity) ratio (PISL) is given by the transmit signal’s time × bandwidth product [1]. Such concept can be associated to the Central Limit Theorem which states that the sum of N independent random variables tends toward a Gaussian distribution with variance N times smaller than the original samples variance, independently of their distribution. Therefore, assuming that for large values of τ, the correlation procedure can be interpreted as a sum of $N=B_{\tilde{s}}\tau_{\tilde{s}}$ independent random variables [12], and that the variance of the transmit signal’s samples is given by their power, 2P, it is possible to write $\mathrm{PISL}=B_{\tilde{s}}\tau_{\tilde{s}}$.

Despite serving as a good approximation, the previous statement can be questioned under two aspects. First, the number of summed samples is only $N=B_{\tilde{s}}\tau_{\tilde{s}}$ when the sampling frequency is equal to the transmit signal’s bandwidth. Second, the independence between the summed samples is only guaranteed if they are Gaussian with rectangular power spectral density. With respect to the former, the authors in [13] introduced the number of effective uncorrelated samples concept to justify the referred approximation. Nevertheless, with respect to the samples independence, it may be necessary to consider a Central Limit Theorem for dependent random variables [38], introducing a higher amount of complexity in the analysis. In the present work, the aforementioned approximation is not considered. Instead, closed form expressions characterizing the PISL are derived and analyzed. Let us then investigate (25) more carefully. First, note that, according to the Chebyshev integral inequality, it is possible to write

(26) $$\begin{array}{c} \int_{-\infty }^{\infty }tri\left(\gamma /\tau_{s}\right)R_{s}^{2}\left(\gamma \right)e^{-j2\pi f_{d}\gamma }d\gamma \geq \int_{-\infty }^{\infty }R_{s}^{2}\left(\gamma \right)d\gamma \cdot \int_{-\infty }^{\infty }tri\left(\gamma /\tau_{s}\right)e^{-j2\pi f_{d}\gamma }d\gamma \end{array}$$

In turn, using the Cauchy–Schwarz inequality for integrals,

(27) $$\begin{array}{c} \int_{-\infty }^{\infty }R_{s}^{2}\left(\gamma \right)d\gamma \geq {\left[\int_{-\infty }^{\infty }R_{s}\left(\gamma \right)d\gamma \right]}^{2} \end{array}$$

Since, according to the Wiener–Khinchine theorem, the signal’s autocorrelation function is the Fourier transform of its power spectral density S(f), replacing (26) and (27) in (25), it is finally possible to write the lower bound for the ISL as

(28) $$\begin{array}{c} E{\left[|\chi_{\tilde{s}}\left(\tau ,f_{d}\right){|}^{2}\right]}_{ISL}\geq \tau_{s}S{\left(0\right)}^{2}{sinc}^{2}\left(f_{d}\tau_{s}\right) \end{array}$$

Now, let us reexamine (25) from a different perspective. Note that (25) is the Fourier transform of a product between two functions, which results in the convolution of their individual Fourier transform as

(29) $$\begin{array}{c} E{\left[|\chi_{\tilde{s}}\left(\tau ,f_{d}\right){|}^{2}\right]}_{ISL}=\tau_{s}F\left[R_{s}^{2}\right]{|}_{f_{d}}*{sinc}^{2}\left(f_{d}\tau_{s}\right) \end{array}$$

where * and F[·] are the convolution and Fourier transform operator, respectively. Since the Fourier transform of the squared autocorrelation function as a function of $f_{d}$ has a much slower decay than ${\mathrm{sinc}}^{2}\left(f_{d}\tau_{s}\right)$, (29) approaches [24]

(30) $$\begin{array}{c} E{\left[|\chi_{\tilde{s}}\left(\tau ,f_{d}\right){|}^{2}\right]}_{ISL}\approx \tau_{s}\pi F\left[R_{s}^{2}\right]{|}_{f_{d}=0} \end{array}$$

Therefore, under the assumptions made, the Doppler shift does not influence the stationary sidelobe level at the matched filter output when random phase or frequency modulated waveforms are employed. This result is particularly interesting, since the main lobe of the PM/FM Noise Radar ambiguity function (see (23)) decreases with the increase of the Doppler frequency while the stationary side lobes level remains the same and independent of τ, i.e., spread in the entire range-Doppler spectrum.

It is important to highlight that the expressions derived in (23) and (30) are valid despite of the stochastic properties of the modulating signal, as long as it is Wrapped Normal. Generally, the mean side lobe level quickly converges to (30), but depending on the latter, a third region appears in (20), which in the present work we characterize as a *transient region*, similar to the one addressed in [14]. Such transient region, if it exists, presents the higher variance of (20), and, unlike in [14], where it was defined as a “deterministic” region, in the present work it also presents a stochastic nature, being responsible for the random peak side lobe (PSL) and not being treatable with windows functions.

When the stationary side lobes level, or the peak side lobe level are unbearable for the specific radar application, optimization procedures are employed to reduce either of them, e.g., [19]. Optimization procedures change the modulating signal properties, in order to reduce this transient region below (30), reducing the integrated side lobes level of the system. Deriving closed form expressions for this specific region under this assumption is not an easy task to perform, being very dependent on the optimization approach adopted. Nevertheless, it is possible to infer the influence of the Doppler shift on it. Let us rewrite (20) as

(31) $$\begin{array}{c} E\left[|\chi_{\tilde{s}}\left(\tau ,f_{d}\right){|}^{2}\right]=\;\;\int_{-\infty }^{\infty }\;\;\;\;\;\;tri\left(\gamma /\tau_{s}\right)g\left(\tau ,\gamma \right)e^{-j2\pi f_{d}\gamma }d\gamma \end{array}$$

Then, as previously done, (31) can also be rewritten as

(32) $$\begin{array}{c} E{\left[|\chi_{\tilde{s}}\left(\tau ,f_{d}\right){|}^{2}\right]}_{TR}=G\left(\tau ,f_{d}\right)*{sinc}^{2}\left(f_{d}\tau_{s}\right) \end{array}$$

where $G(\tau ,f_{d})$ is the Fourier transform of g(τ,γ). When optimizing random waveforms for null Doppler shifts, (32) indicates that targets with velocity will present higher sidelobes at the output of the matched filter and that the latter is more critical for long pulses.

**Figure 1:**
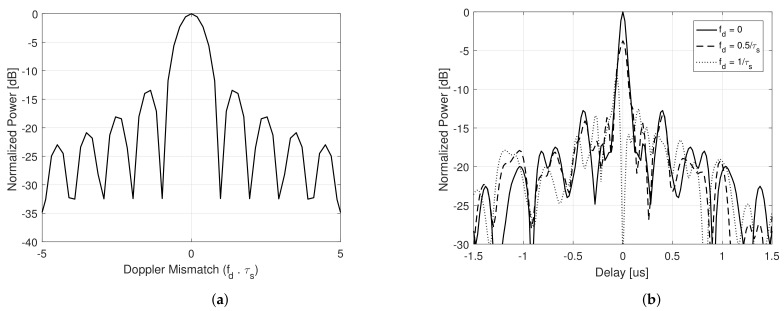
(**a**) Doppler profile (range cut) and (**b**) range profile (Doppler cut).

Figure 1 respectively illustrates the Doppler profile and range profile cuts (considering different values of Doppler shifts—$f_{d}=0$, $f_{d}=0.5/\tau_{s}$ and $f_{d}=1/\tau_{s}$) of the Ambiguity Function for a single pulse realization of $\tilde{s}\left(t\right)$, considering a transmit signal with a bell shaped autocorrelation function. Note that the main lobe behavior is precisely the one described by (23), vanishing for $f_{d}=1/\tau_{s}$, while the side lobes, even though exhibiting a random behavior, present their mean value according to (30), invariant with the Doppler shift [27]. In obtaining the referred images, it was considered $\tau_{s}=10\;\mu$s and $B_{\tilde{s}}=10$ MHz, but the same behavior is expected despite the parameters used.

Since the Doppler tolerance of PM/FM Noise waveforms are independent of their range profile, the remainder of this paper addresses only the latter. The system’s performance, being a function of $R_{\tilde{s}}\left(\tau \right)$, is also function of $R_{\theta }\left(\tau \right)$. The autocorrelation function of the random process characterizing the phase of the transmit signal, according to the Wiener–Khintchine theorem, is obtained by the Fourier transform of its Power Spectral Density, $S_{\theta }\left(f\right)$, which, in turn, is given by

(33) $$\begin{array}{c} S_{\theta }\left(f\right)={\left|H\left(f\right)\right|}^{2}S_{a}\left(f\right) \end{array}$$

where H(f) is a given filter transfer function and $S_{a}\left(f\right)$ is the modulating signal power spectral density. Therefore, both the modulating signal power spectral density as well as the transfer function of a given filtering operation performed on the latter are extremely important to the Noise Radar performance. In the following sections, the aforementioned parameters are addressed in more depth. The outcome of (20) is closely investigated under different assumptions of the stochastic properties of the modulating signal and filtering operation. More precisely, the power spectral density and variance of the modulating signal are varied and the expected values of the corresponding matched filter output powers are compared. With respect to the filtering operation, only phase and frequency modulation are taken into consideration. More complex filtering operations are out of the scope of the present work.

### 3.1. Random Frequency Modulated Signal

In [8,39], the outcome of (20) was analyzed in detail, considering a modulating random process a(t) with flat power spectral density over a frequency range of $2B_{a}$, $S_{a}\left(f\right)={\mathrm{rect}}_{B_{a}}\left(f\right)$, and frequency modulation, i.e.,

(34) $$\begin{array}{c} H\left(f\right)=\left[\frac{1}{2}\delta \left(f\right)+\frac{1}{j2\pi f}\right] \end{array}$$

The frequency modulated transmit signal autocorrelation function is thus given considering (14), (33) and (34) by

(35) $$\begin{array}{c} R_{\tilde{s}}\left(\tau \right)=2Pe^{-\left[\sigma_{a}^{2}\frac{1}{4\pi^{2}B_{a}}\int_{0}^{B_{a}}\frac{1-\cos(2\pi f\tau )}{f^{2}}df\right]} \end{array}$$

where $\sigma_{a}$ is the standard deviation of the random samples that characterize the modulating signal.

When $\sigma_{a}\gg B_{a}$, it has been shown in [8] that $R_{\tilde{s}}\left(\tau \right)$ does not depend on the modulating signal’s bandwidth and (35) approaches the Gaussian shape

(36) $$\begin{array}{c} R_{\tilde{s}}\left(\tau \right){|}_{NBFM\;rect}=2Pe^{-\sigma_{a}^{2}\tau^{2}/2} \end{array}$$

For the sake of completeness the matched filter output 3 dB width, the power spectral density and the 90% bandwidth relative to the aforementioned transmit waveform, derived in [8] are presented hereafter. The 3 dB width of the matched filter output (see (23)), that leads to the system range resolution is given by

(37) $$\begin{array}{c} \Delta \tau_{3dB}{|}_{NBFM\;rect}=\frac{2\sqrt{\ln2}}{\sigma_{a}} \end{array}$$

As previously mentioned, according to the Wiener–Kintchine theorem, the power spectral density $S_{\tilde{s}}\left(f\right)$ of the transmit signal’s complex envelope is given by the Fourier transform of its autocorrelation function, which results in

(38) $$\begin{array}{ccc} S_{\tilde{s}}\left(f\right){|}_{NBFM\;rect} & = & \frac{2P\sqrt{2\pi }}{\sigma_{a}}e^{-\left[\frac{2\pi^{2}f^{2}}{\sigma_{a}^{2}}\right]} \end{array}$$

The analysis of the transmit signal’s power spectral density allows the evaluation of its spectral containment as well as the derivation of closed form expressions describing its bandwidth. In the present work, two widespread procedures to evaluate a signal’s bandwidth are considered, the 3 dB bandwidth and the portion of the spectrum where *p* % of total power is concentrated. The latter is proved to be

(39) $$\begin{array}{c} B_{\tilde{s}-p\%}{|}_{NBFM\;rect}=Q^{-1}\left(\frac{1-p}{2}\right)\frac{\sigma_{a}}{\pi } \end{array}$$

where $Q^{-1}\left(x\right)$ is the inverse of the Q-function [26]. The 3 dB bandwidth, in turn, is given by

(40) $$\begin{array}{c} B_{\tilde{s}-3dB}{|}_{NBFM\;rect}=2\sqrt{2\ln2}\frac{\sigma_{a}}{2\pi } \end{array}$$

Considering (23), (29) and (36), for the zero Doppler special case, the peak to stationary side lobe (or side lobe at infinity—ISL) ratio is evaluated as

(41) $$\begin{array}{c} PISL{|}_{NBFM\;rect}=\frac{\tau_{s}\sigma_{a}}{\sqrt{\pi }} \end{array}$$

Note that (41) can be rewritten in terms both of $B_{\tilde{s}-p\%}$, as well as $B_{\tilde{s}-3dB}$ (see (39) and (40)). Therefore, despite serving as a good approximation, the peak to stationary side lobe level ratio is not simply given by the transmit signal’s time × bandwidth product.

As an alternative to the narrowband frequency modulation described, wideband frequency modulation is obtained when high values of $B_{a}$ are considered. In such case, (35) approaches

(42) $$\begin{array}{c} R_{\tilde{s}}\left(\tau \right){|}_{WBFM\;rect}=2Pe^{-\left[\frac{\sigma_{a}^{2}\left|\tau \right|}{4B_{a}}\right]} \end{array}$$

The 3 dB width of the matched filter output (see (23)) is given by

(43) $$\begin{array}{c} \Delta \tau_{3dB}{|}_{WBFM\;rect}=\frac{4B_{a}\ln2}{\sigma_{a}^{2}} \end{array}$$

The power spectral density, in turn, is the Fourier transform of a two-sided decaying exponential, which results in a Lorentzian function written as

(44) $$\begin{array}{c} S_{\tilde{s}}\left(f\right){|}_{WBFM\;rect}=\frac{\frac{2P\sigma_{a}^{2}}{2B_{a}}}{\frac{\sigma_{a}^{4}}{16B_{a}^{2}}+{\left(2\pi f\right)}^{2}} \end{array}$$

Similarly to what has been previously done, two calculations of bandwidth are considered. First, the *p*% bandwidth is calculated as

(45) $$\begin{array}{c} B_{\tilde{s}}{|}_{WBFM\;rect-p\%}=\frac{\tan\left(p\frac{\pi }{2}\right)}{\pi }\frac{\sigma_{a}^{2}}{4B_{a}} \end{array}$$

The 3 dB bandwidth is then given by

(46) $$\begin{array}{c} B_{\tilde{s}}{|}_{WBFM\;rect-3dB}=\frac{1}{\pi }\frac{\sigma_{a}^{2}}{4B_{a}} \end{array}$$

Again, considering (23), (29) and (42), for the zero Doppler special case, the peak to stationary side lobe ratio is evaluated as

(47) $$\begin{array}{c} PISL{|}_{WBFM\;rect}=\frac{\tau_{s}\sigma_{a}^{2}}{4B_{a}} \end{array}$$

Note that, similarly to the narrowband frequency modulation, (47) can also be rewritten in terms both of $B_{\tilde{s}-p\%}$, as well as $B_{\tilde{s}-3dB}$. Nevertheless, unlike the former, where the transmit signal’s 3 dB bandwidth and 90% bandwidth are closely related (see (39) and (40)), when considering wideband frequency modulated signals, such relation differs considerably (see (45) and (46)). Therefore, the relation of both the 3 dB width of the matched filter output as well as the peak to stationary side lobe ratio will have significantly distinct values depending on the standardization employed.

**Figure 2:**
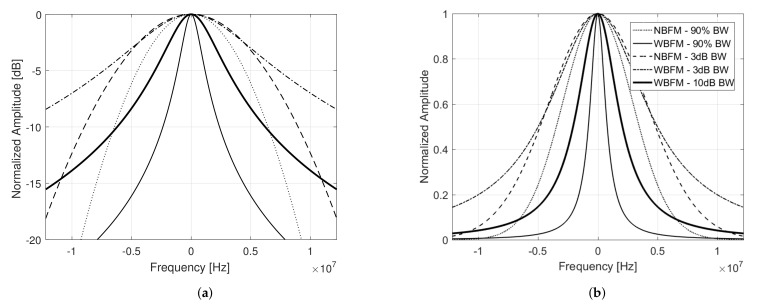
Power spectral density of narrowband (NBFM) and wideband (WBFM) frequency modulated signals with 10μs duration, plotted (**a**) in dB and (**b**) in linear scale, considering different combinations of $\sigma_{a}$ and $B_{a}$ that result in bandwidths $B_{\tilde{s}}=10$ MHz under different criterion.

**Figure 3:**
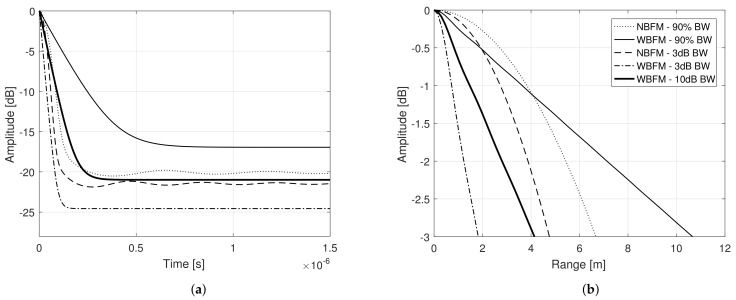
(**a**) Matched filter output powers and (**b**) their corresponding 3 dB widths brought on focus of narrowband (NBFM) and wideband (WBFM) frequency modulated signals with 10μs duration, considering different combinations of $\sigma_{a}$ and $B_{a}$ that result in bandwidths $B_{\tilde{s}}=10$ MHz under different criterion.

Figure 2 and Figure 3 respectively illustrate the power spectral density and the matched filter output power of narrowband and wideband frequency modulated signals with 10μs duration, considering different combinations of $\sigma_{a}$ and $B_{a}$, that result in bandwidths $B_{\tilde{s}}=10$ MHz under the aforementioned definition criterion. The matched filter output power 3 dB widths are brought on focus, where the x-axis are converted from time τ to range R=cτ/2, with *c* being the vacuum light speed.

Note that the requirements relative to the transmit signal’s spectral containment directly impacts the system’s range resolution and peak to stationary side lobe level. Therefore, properly defining the former can lead to systems with enhanced performance. It is important to highlight that the transmit signal’s bandwidth definition has also to take into consideration the radar transmitter limitations. Spectral leakage and attenuation of frequency components outside the specified bandwidth are examples of side effects that appear in systems that do not properly address such issue [17]. More specifically to the latter, frequency component attenuation introduces amplitude modulation effects to the signals involved (transmitted and received), possibly distorting the matched filter output as well, as will be shown later on.

In general, when Gaussian modulating signals are taken into consideration, FM Noise Radar system performance is ruled by relatively simple closed form expressions. Nevertheless, frequency modulation, if performed in real time, requires a specific amount of resources that may not always be available. Therefore, phase modulation may sometimes be preferred. In the following section such subject is addressed in more depth.

### 3.2. Random Phase Modulated Signal

In the present section, we extend the performed study considering phase modulation, i.e., H(f)=1 (see (33)) and varied modulating signal’s properties. First, let us assume that the modulating process a(t) also presents flat power spectral density over a frequency range of $2B_{a}$. Then, $R_{\tilde{s}}\left(\tau \right)$ is given by

(48) $$\begin{array}{c} R_{\tilde{s}}\left(\tau \right){|}_{PM\;rect}=2Pe^{-\sigma_{a}^{2}\left(1-sinc\left(\pi 2B_{a}\tau \right)\right)} \end{array}$$

The following modulating signal we address presents triangular power spectral density over a frequency range of $2B_{a}$. The transmit signal’s autocorrelation function is thus

(49) $$\begin{array}{c} R_{\tilde{s}}\left(\tau \right){|}_{PM\;tri}=2Pe^{-\sigma_{a}^{2}\left(1-sinc{\left(\pi 2B_{a}\tau \right)}^{2}\right)} \end{array}$$

Finally, the last modulating signal taken into consideration in the current analysis presents a Gaussian shaped power spectral density over a frequency range of $2B_{a}$, given by

(50) $$\begin{array}{c} S_{a}\left(f\right)=\left\{\begin{array}{ccc} \frac{1}{B_{a}\sqrt{\pi }}e^{-\frac{f^{2}}{B_{a}^{2}}} & ; & \left|f|\leq \right|B_{a}|\\ 0 & ; & \left|f|>\right|B_{a}|. \end{array}\right. \end{array}$$

The transmit signal’s autocorrelation function is thus

(51) $$\begin{array}{c} R_{\tilde{s}}\left(\tau \right){|}_{PM\;gauss}=2Pe^{-\sigma_{a}^{2}\left(1-e^{-{\left(\pi B_{a}\tau \right)}^{2}/2}\right)} \end{array}$$

The derivation of closed form expressions relative to the matched filter output power (20) for each of the aforementioned PM signals is out of the scope of the present work. Nevertheless, it is possible to note that all modulating signals addressed will lead to PM transmit signals with bell shaped autocorrelation functions. Ideally, according to (3), they should then present similar behavior when employed in Noise Radar systems, but instead, they present considerably different outcomes of (20), that should be properly compared in order to define the most appropriate waveform generation method for radar applications.

**Figure 4:**
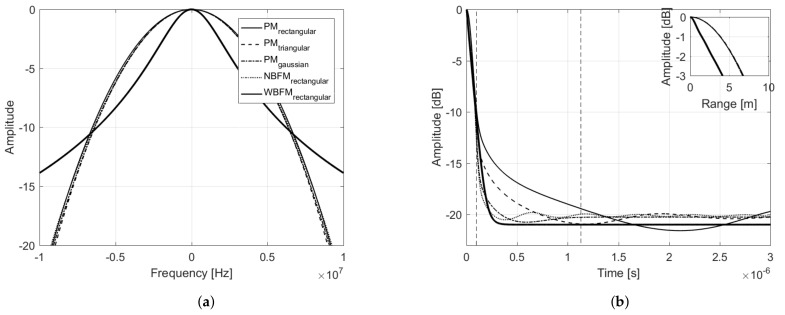
(**a**) Power spectral density and (**b**) Matched filter output power of different transmit signals obtained varying the modulating signal’s properties and the modulation procedure.

Figure 4a presents the power spectral density of each transmit signal, considering the aforementioned modulating signals and phase modulation. In order to perform an unbiased analysis, $B_{a}$ and $\sigma_{a}$, for each modulating signal, were chosen in such a way as to ensure that all corresponding transmit signals had the same bandwidth. In obtaining the following results, it was considered $\tau_{s}=10\;\mu$s and $B_{\tilde{s}}=10$ MHz. For comparison purposes, narrowband and wideband frequency modulation were also introduced in the analysis, considering the 90% bandwidth concept (see (39) and (45)). Note that all PM transmit signal’s power spectral densities present a very similar bell shape. The outcomes of (20), considering each of the described modulating signal, are presented in Figure 4b, where their 3 dB width are brought on focus, with the x-axis converted from time τ to range R=cτ/2, with *c* being the vacuum light speed.

**Figure 5:**
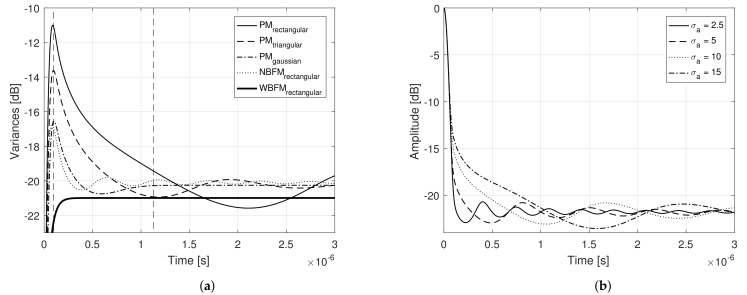
(**a**) Variance of the matched filter output power relative to different transmit signals obtained varying the modulating signal’s properties and the modulation procedure and (**b**) expected value of the matched filter output power of different transmit signals obtained varying the modulating signal’s random samples standard deviation and bandwidth, considering a rectangular power spectral density and phase modulation.

Note that they all present a bell shape and the main lobe characterization is nearly the same for all. Such behavior leads to the conclusion that, if the transmit signals present similar bandwidth, they will also present similar range resolutions, independently of the modulating signal. The same remark is valid for the stationary side lobe level. Nevertheless, as previously mentioned, a third region appear in (20), which in the present work we characterize as a *transient region*. Figure 5a presents the variance of the matched filter output, or the equivalent variance of the ambiguity function, given by

(52) $$\begin{array}{c} \sigma^{2}=E\left[|\chi_{\tilde{s}}\left(\tau ,f_{d}\right){|}^{2}\right]-E{\left[|\chi_{\tilde{s}}\left(\tau ,f_{d}\right)|\right]}^{2} \end{array}$$

where $E\left[|\chi_{\tilde{s}}\left(\tau ,f_{d}\right){|}^{2}\right]$ is given according to (20) and $E\left[|\chi_{\tilde{s}}\left(\tau ,f_{d}\right)|\right]$ is defined by (3).

Figure 5a shows that such transient region presents the higher variance of the matched filter output, i.e., it is the region with the greater dispersion of (20), or, equivalently, the region with the higher squared deviation from its mean value (3) and, therefore, responsible for the random peak side lobe (PSL). Unlike what has been done in [14], where deterministic sidelobes are reduced with the employment of window functions that conveniently shape the Power Spectral Density (PSD) of amplitude modulated random transmit signals, the PSL that arises from the transient region under study in the present work is random and not related to the PSD shape.

This transient region is dependent on the modulating signal’s properties and can significantly compromise the system performance, if not properly taken into consideration. The narrowband frequency modulated signal is the one that presents a smaller (sharper) transient region and thus, smaller peak side lobe levels are expected. On the other hand, the considered phase modulated signal with rectangular power spectral density is the one that presents a higher (and longer) transient region and hence higher peak side levels are expected. Nevertheless, such transient region is not only a function of the shape of the modulating signal’s power spectral density, but it is also a function of $B_{a}$ and $\sigma_{a}$. Figure 5b presents the matched filter output power, considering phase modulation and modulating signals with rectangular power spectral density, for different $B_{a}$ and $\sigma_{a}$. Note that the transient region increases as the variance of the modulating signal’ random samples decreases.

### 3.3. Simulation

**Figure 6:**
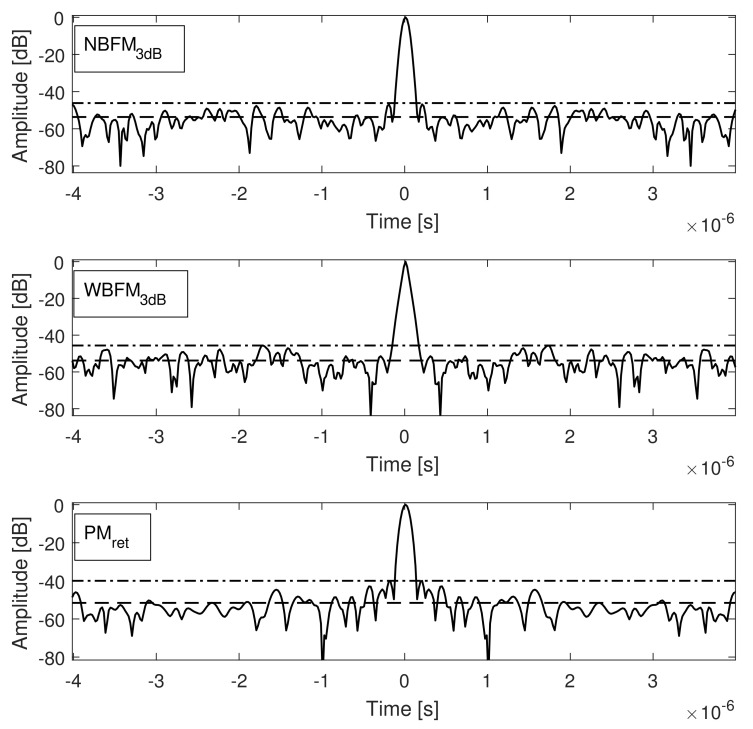
Comparison between matched filter output powers, considering both PM, narrowband, and wideband FM (3 dB Bandwidth) random modulated signals and Gaussian modulating signals with rectangular power spectral density—$B_{\tilde{s}}=10$ MHz, $\tau_{s}=10\;\mu$s, $n_{p}=1000$.

In the present section, software simulations were performed to verify the robustness of the analytical conclusions drawn in the previous section. It was considered modulating signals, a(t), with flat power spectral density over a frequency range of $2B_{a}$, both phase modulated as well as frequency modulated (narrowband and wideband). Figure 6 presents a comparison between the matched filter output power when PM, narrowband FM, and wideband FM transmit signals, considering the 3 dB Bandwidth concept described in the previous section, are employed. In obtaining Figure 6, $n_{p}=1000$ realizations of a(t) and, as a consequence, $\tilde{s}\left(t\right)$, were evaluated and their corresponding matched filter outputs, for each generation procedure, were summed, in an attempt to better characterize their expected values. Due to the stochastic nature of the matched filter outputs, their side lobes may vary for each realization, but their mean values are given according to the closed form expressions derived throughout the present work.

The matched filter outputs main lobe present a relative small variance; therefore, its behavior remains nearly the same despite the modulating signal realization (given according to (23)). Stationary side lobes of the matched filter outputs, due to their higher variance, are different for distinct transmit signal realizations, i.e., they randomly oscillate around their expected mean value (given according to (30)). Finally, note that the peak side lobe also presents a random behavior; nevertheless, in accordance to the analysis performed in the previous section, it is considerably higher for the phase modulated signal.

### 3.4. Evaluation of Randomness

Now, let us address the degree of randomness of PM/FM Noise Radar transmit signals, considering Wrapped Normal modulating signals. It is a known fact within the Noise Radar community that one of the major advantages of such systems is their low probability of intercept (LPI), introduced by the employment of pseudo-random waveforms. At this point, it is important to highlight that Noise Radars are still susceptible of being detected by Electronic Intelligence (ELINT) systems using traditional methods, such as the analysis of coherency (in multiple inputs systems) and time (specially for pulsed radars) and frequency thresholding [40]. Furthermore, they may also suffer electronic attacks by systems able to generate deception jamming in real time, just as classical radars that employ deterministic transmit waveforms. Therefore, classical electronic countermeasures such as frequency agility, varied pulse repetition frequencies (in pulsed radar), and low transmit power remain as good design practices in Noise Radars.

Thus, what enhances the LPI characteristic of a Noise Radar system is their ability to pose as thermal noise for less sophisticated ELINT systems, remaining covert, and most importantly, their ability to generate varied sample functions of the same stochastic process, inhibiting the action of deception systems that do not operate in real time. Such features are related to the transmit waveforms degree of randomness, that should be properly analyzed. In [23], the authors investigated the Mutual Information Rate (MIR) of each transmit signal considered, stating that the analysis of the waveform’s additional information obtained when one more sample is observed is fundamental to define its Low Probability of Intercept (LPI) characteristic. In the referred work, the authors evaluated both the Spectral Flatness Measure (SFM) of such waveforms, as well as the MIR, which is related to the entropy rate of complex stochastic processes [41,42], and measures the information increase as a function of time. For Gaussian processes [43], the SFM is directly related to the MIR, and is given, by

(53) $$\begin{array}{c} SFM=\frac{\exp\left[\int_{-\infty }^{\infty }\ln\left[S\left(f\right)\right]df\right]}{\int_{-\infty }^{\infty }S\left(f\right)df} \end{array}$$

Values of SFM close to 1 indicate a random signal, while values close to 0 evidence a well structured signal. Despite serving as a good measure of randomness, (53) can lead towards incorrect conclusions when the waveform under study presents several frequencies with null or approximately null components [44], or when non-Gaussian processes are under study [43], inciting the search for other measures that can contribute for the analysis.

In [14], it is reported an ongoing work that is being developed by the same group of authors to evaluate the degree of randomness of amplitude modulated pseudorandom waveforms for Noise Radar application, which had not been published by the time of submission of the present work. Therefore, and in the absence of a common sense of the scientific community on how the randomness of transmit waveforms should be evaluated for Noise Radar applications, in the present work we evaluate the SFM of different narrowband frequency modulated signals addressed and also compare their performance, with respect to randomness, to what can be considered a true random signal. The latter is achieved by using the Statistical Test Suite for Random and Pseudorandom Number Generators for Cryptographic Applications [45] from the National Institute of Standards and Technology (NIST) of the U.S. Department of Commerce. The narrowband FM signal was chosen for the analysis because it presents the better tradeoff between spectral confinement and side lobe level, and due to its similar properties obtained when considering a 3 dB bandwidth and a 90% power bandwidth.

The Noise waveforms were generated over a sampling frequency of 100 MHz, considering 3 dB bandwidths $B_{\tilde{s}}=\left[3,10,20,50\right]$ MHz and $\tau_{s}=20.48$ μs duration. This procedure generates a vector of 2048 samples, each one of them 16 bits long. Others 255 were also produced in order to acquire a bigger set for the randomness test. The True Random Noise was generated by the use of the python library *rdrand* [46] that uses the thermal noise of the computer’s processor in order to output a true random bit. The generator used was RdRandom() and it was produced the same amount of bits as the noise waveform, that is, $2^{23}$ bits. All the standard configurations of the test suite were kept the same. All the tests were run for the 5 datasets for bitstreams of 32,768 bits for 256 runs.

The test suite is composed by several tests, with little redundancy between them, i.e., each test is able to detect specific pattern behaviors. In the present work, and considering a Noise Radar application, we evaluate the described FM pseudo-random waveforms with respect to the number of occurrences of “1” s and “0” s in their binary representation (Frequency test), which should be nearly the same, the linear dependence among fixed subsamples of the original datasets (Rank test) and the presence of periodic features (repetitive patterns) within the datasets (FFT test, Entropy test and Serial test). A detailed description of the performed tests is out of the scope of the present work, for that purpose, the reader is advised to go to [45].

**Figure 7:**
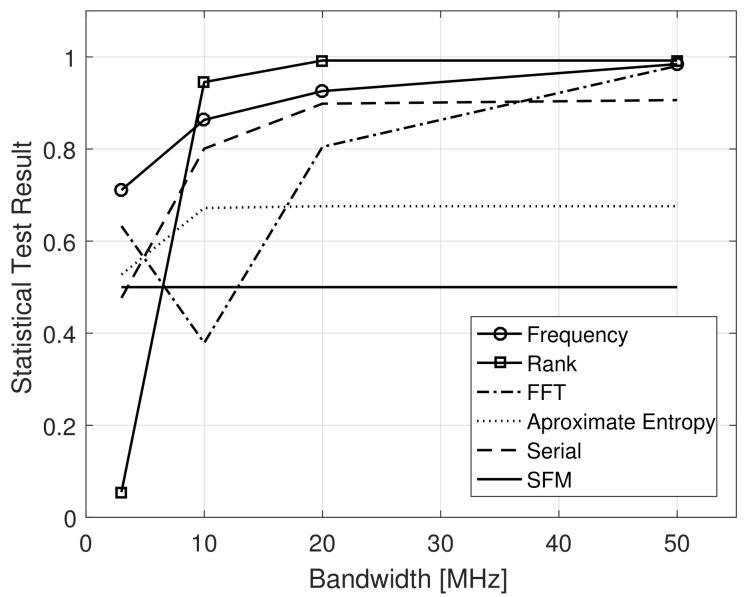
Statistical test results considering 4 pseudorandom narrowband frequency modulated signals (${\mathrm{NBFM}}_{3\mathrm{dB}}$) as a function of their 3 dB bandwidth—3 MHz, 10 MHz, 20 MHz, and 50 MHz.

Figure 7 presents the statistical results of the aforementioned tests along with the Spectral Flatness Measure for the previous described narrowband FM pseudo-random signals as a function of their 3 dB bandwidth. Note that, contrary to the SFM that presents a nearly constant behavior, indicating a good degree of randomness, the statistical tests from NIST show a high dependency on the FM transmit signal’s bandwidth. A convergence behavior can also be observed, from the tests, indicating that signals with 10 MHz bandwidth and above already present a performance with respect to randomness close to the one obtained by true random numbers generators.

Once again, it is important to highlight that good performance with respect to statistical tests for randomness do not necessarily implicate that the next sample of a given pseudo-random waveform is unpredictable, given their previous values (forward unpredictability) [45], nor that the signals are undetected by ELINT systems. To achieve the former, more complex analyses on both the pseudorandom generator, as well as on the statistics of the datasets need to be performed, while for the latter, there is a need to fully cover detectors based on the statistics of the transmit signals, like the ones proposed in [47]. Both studies are out of the scope of the present work.

### 3.5. Spectral Containment—Hardware Evaluation

The previous sections highlighted the importance in properly defining the stochastic properties of the modulating signal in order to obtain transmit signals with desired power spectral densities and corresponding autocorrelation functions. Nevertheless, the choice of the aforementioned parameters has also to take into consideration the system’s hardware constraints. As previously shown, bell shaped autocorrelation functions lead to bell shaped power spectral densities, which are not well contained, i.e., a considerable portion of the spectrum may lay outside of the specified bandwidth. Therefore, the hardware components in the transceiver chain may introduce distortions to the received signals, leading to unavoidable undesired effects.

There are two main reasons for that: the shape of the Power Spectral Density (PSD) and its randomness. As previously discussed, the PSD is usually designed to be bell shaped (due to its relation to the autocorrelation function); therefore, a considerable portion of the spectrum can be outside the RF filters pass band. Regarding the latter, due to the stochastic nature of the signals involved, the received signal’s PSD, likewise the side lobes of the matched filter output, also present a random behavior. Therefore, even though their expected values are properly characterized, variations between transmit signals are expected, and, unavoidably, some of them will present a higher bandwidth than the specified, being more susceptible to distortions.

In the present section, we take into consideration a 48 channel S-band phased array prototype (PO1) to evaluate possible impacts of hardware distortion on the previously analyzed waveforms and verify the robustness of the analytical conclusions drawn throughout the present work. The PO1, is a radar developed for research and development by the Brazilian Army, with very traditional RF chains, consisting in digital to analog (DAC) converters, filters, mixers, transmit amplifiers, low noise amplifiers, and analog to digital converters (ADC). The signal processing of the received signal is also classic, consisting in Digital Down Converters (DDC) with digital filtering, matched filtering, pulse integration, and a CFAR detector, all performed on a proprietary board split between two Virtex 5 FPGAs. Since we did not want to introduce undesired variables to the analysis, we used a simple setup with a loopback connection prior to the T/R modules, instead of a transmission and capture of real echos, which would invariably come with a huge amount of clutter and interferences added.

As in the previous section, the bench top test was performed using the narrowband frequency modulated signal, since it presents the best tradeoff between spectral confinement and side lobes level and due to its similar properties obtained when considering a 3 dB bandwidth and a 90% power bandwidth. The duration of the transmit signal was set to $\tau_{s}=20.48\;\mu$s, and the bandwidth (considering the corresponding criterion) was established at $B_{\tilde{s}}=3$ MHz.

**Figure 8:**
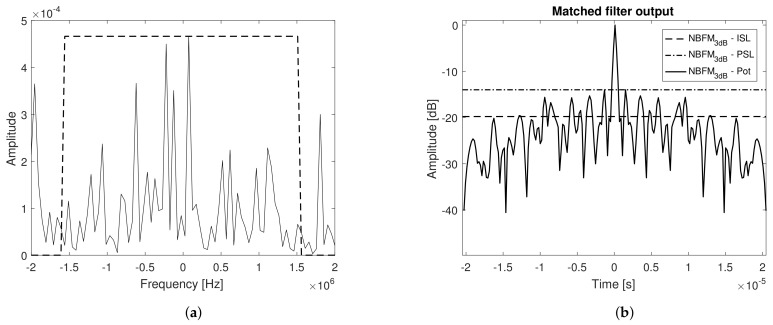
Power Spectral Density (**a**) and matched filter output power (**b**) of a pseudorandom narrowband frequency modulated signal realization (${\mathrm{NBFM}}_{3\mathrm{dB}}$)—$B_{\tilde{s}}=3$ MHz, $\tau_{s}=20.48\;\mu$s.

The power spectral density and matched filter output power of the above mentioned waveform, prior to digital to analog conversion, are presented in Figure 8a,b, respectively.

**Figure 9:**
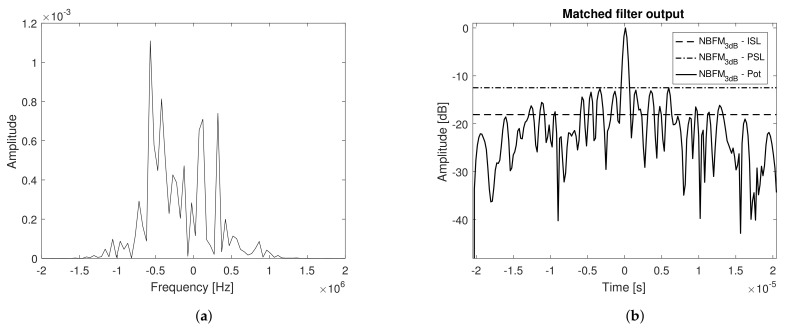
(**a**) Power Spectral Density (PSD) and (**b**) matched filter output power of the loopback signal, considering a narrowband frequency modulated random transmit signal (${\mathrm{NBFM}}_{3\mathrm{dB}}$).

Note that the theoretical values of bandwidth, resolution, and peak to stationary side lobe value (PISL) are in accordance to the ones derived in Section 3.1. In Figure 8b, the Peak to Peak Side Lobe value (PSL) is also presented. The power spectral density of the received signal (loopback connection), prior to pulse compression, and the corresponding matched filter output power are depicted in Figure 9a,b, respectively.

The transmit and receive paths introduce distortions to the received signals, as noted from their power spectral densities analysis. The attenuation of frequency components outside the specified bandwidth (3 MHz) is evident, which causes not only an unavoidable mismatch loss but also distortions to the matched filter output inducing not only lower values of PISL but also higher values of peak side lobe level and a wider matched filter output 3 dB width, resulting in worse resolution. All three drawbacks can compromise the system’s overall performance; nevertheless, the higher side lobes, especially in the vicinity of the main lobe (PSL), are specially critical, since they are not foreseen in the theoretical formulation for this type of waveform. It is possible to conclude that the attenuation of frequency components outside the specified bandwidth may be critical to Noise radar systems, introducing different distortions that can impact the matched filter performance.

### 3.6. FPGA Design and Implementation

Another important characteristic of pseudo-random waveforms for Noise Radar applications that has started attracting the attention of the scientific community in the past few years is their feasibility of real time generation, without the usage of an external hardware source, while retaining the properties specified by their model. The latter increases the LPI performance of such radars and suits better cognitive systems. The computational burden to implement optimization algorithms in real time applications is seldom bearable, i.e., optimized waveforms are generally created offline and stored in on board memories, limiting their range of applications. Furthermore, modern radar systems that require real-time signal processing, including waveform generation, generally make use of Field Programmable Gate Arrays (FPGAs), which, unlike application specific integrated circuit (ASIC) that are generally designed for a single purpose, are programmable integrated circuits, that add flexibility, scalability, low power consumption and reliability to the design. In [48], for example, a linear congruential algorithm implemented in FPGA is taken into consideration to generate pseudo-random sequence samples for Noise Radars. Despite being a real time approach, the solution proposed in [48] generates amplitude modulated waveforms, which do not present high power efficiency, as previously mentioned.

Gaussian distributed, band limited pseudo-random modulating signals are the basis to all derivations considered in the present work. A simple way to generate such type of signals is with the Box–Muller transform, considering two uniformly distributed random numbers, $u_{1}\left(t\right)$ and $u_{2}\left(t\right)$ [49], i.e.,

(54) $$\begin{array}{c} a_{B\;M}\left(t\right)=\sqrt{-2\ln(u_{1}\left(t\right))}\cos\left(2\pi u_{2}\left(t\right)\right) \end{array}$$

Pseudo-random sequence samples that present a distribution close to uniform can, in turn, be generated with Linear Feedback Shift Registers (LFSR). LFSRs are electronic circuits, composed by X-OR operations and shift registers that generate linear recurring sequences. Being fast and easy to implement both in software as well as in hardware, LFSR have been successfully employed in cryptography, error correcting codes, and spread spectrum communication [50]. Assuming that two LFSR, with different seeds, are generating the pseudo-random sequences $u_{1}\left(t\right)$ and $u_{2}\left(t\right)$, it is possible to approximate them to uniformly distributed random numbers.

The Box–Muller transform requires natural logarithm, square root and cosine operations being very resource consuming when implemented in hardware, especially considering that Digital Signal Processing blocks (DSPs) are scarce and also widely used in the receiver processing chain. A simpler approach relies on the summation of different LFSRs pseudorandom sequences. Assuming they are uniformly distributed, their summation presents an Irwin–Hall distribution, that can be approximated to a Gaussian distribution when the number of summed sequences tends to infinity. Therefore, within the present work we also consider the modulating signals $a_{2}\left(t\right)=u_{1}\left(t\right)+u_{2}\left(t\right)$ and $a_{4}\left(t\right)=\sum_{i=1}^{4}u_{i}\left(t\right)$ in the analysis, where $u_{3}\left(t\right)$ and $u_{4}\left(t\right)$ are also pseudo-random sequences generated by other two LFSR, with different seeds.

**Figure 10:**
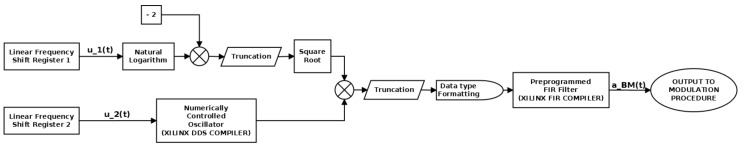
Block diagram of the pseudorandom waveform generation method considering the Box–Muller transform approach in *Xilinx^®^* System Generator.

After generating the above mentioned modulating signals, a FIR filter is still employed, prior to the frequency modulation operation that is performed using the Direct Digital Synthesizer (DDS) compiler IP from Xilinx. The FM pseudo-random waveform generation architectures described are first validated in *Xilinx^®^* System Generator [51], a design tool that enables the use of Xilinx’s IPs inside *MathWorks* model-based *Simulink^®^* design environment for purposes of integration and simulation alongside *Matlab^®^* capabilities for an optimized FPGA implementation. Figure 10 presents the block diagram of a typical implementation for generating the random modulating signals considering the Box–Muller transform approach, used in the present analysis. The frequency modulation procedure has also been implemented in *Xilinx^®^* System Generator, but its diagram is omitted.

The block diagram in Figure 10 is composed by generic IPs from *Xilinx^®^*, e.g., Linear Frequency Shift Registers, Natural Logarithm and Square root, blocks versioned by *Xilinx^®^*, i.e., DDS Compiler 6.0 and FIR Compiler 7.2 and lastly, data preparation blocks to the incoming operation (“Truncation” and “Data Type Formatting”). The approximated overall system’s latency is hence a sum of each individual blocks combined with their needs for data. In the present work we are using a 25 MHz system clock, resulting in a 61 clock cycles latency. Finally, it is important to highlight that the proposed architecture is synchronous, i.e., all the logic is encapsuled between registers. Therefore, there is a need to use additional registers to reduce critical paths, assuring that all transitions are stable before the next rising edge of the clock, meeting all the timing constraints. The generated pseudorandom modulating signals as well as the corresponding frequency modulated transmit signals are captured by specific *Xilinx^®^* System Generator “Gateway Out” blocks and analysed in *Matlab^®^*.

**Figure 11:**
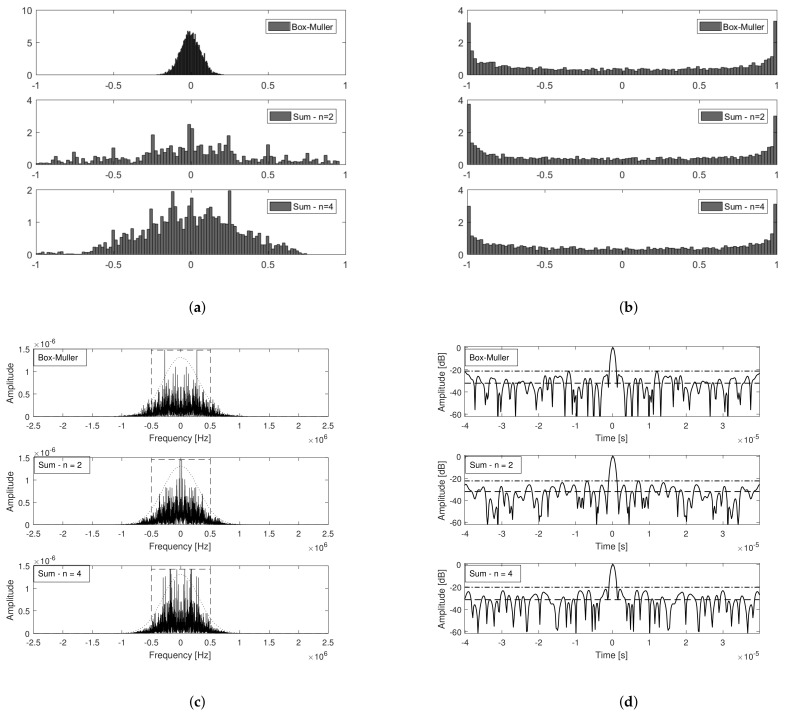
(**a**) Modulating signal’s distributions, (**b**) Transmit signal’s distributions, (**c**) Power Spectral Densities and (**d**) FM Noise pseudo-random waveforms matched filter output (ISL in dashed line and PSL in dot dashed line).

Figure 11a presents the distributions of the modulating signals $a_{B\;M}\left(t\right)$, $a_{2}\left(t\right)$ and $a_{4}\left(t\right)$. As expected, the modulating signal generated using the Box–Muller approach presents a distribution that is closer to the Gaussian. The measured Kurtosis for each dataset was 2.9303 (Box-Muller), 2.7721 (Sum, *n* = 2) and 2.8130 (Sum, *n* = 4), while the measured Spectral Flatness Measure (see (53)) was 0.5751(Box-Muller), 0.5698 (Sum, *n* = 2) and 0.5663 (Sum, *n* = 4). Figure 11b presents the distributions of the corresponding transmit signals generated using each of the modulating signals described. Note that they present nearly the same distributions.

Figure 11c presents the Power Spectral Densities of the narrowband FM noise waveforms generated considering each of the above mentioned modulating signals, a FIR filter with 200 KHz pass band, $\sigma_{a}=2\times 10^{6}$, and $\tau_{s}=1$ ms. Note that they all present a shape close to (38) and 90% energy bandwidth given by (39), which in the present analysis is $B_{\tilde{s}-90\%}{|}_{\mathrm{NBFM}\mathrm{rect}}=1$ MHz. Finally, Figure 11d presents the respective matched filter outputs along with the Stationary Side Lobes (or side lobes level at infinity—ISL) levels and the Peak Side Lobe levels (PSL). Once again, note that the results are all in agreement with the closed form expressions derived in the previous section. With respect to the main lobe and 3 dB resolution, they all follow (23), (36) and (37), respectively. Peak to stationary side lobe levels are also in accordance to (41), i.e., nearly 30 dB. In Figure 11d, the Peak to Peak Side Lobe value (PSL) is also presented for each type of waveform. Being random waveforms, characterized by a stochastic process, side lobes, due to their also random behavior (higher variance), are different between transmit signals, despite presenting the same expected mean value (as derived in Section 3.1). Therefore, the peak value of the side lobes is a quantity difficult to model through closed form expressions. Nevertheless, they pose as interesting features to evaluate when comparing random waveforms performance, since they directly impact the overall system performance.

Figure 11c,d indicate that the modulating signal generation method is not critical, since the resultant FM pseudo-noise waveforms present similar performance under a matched filter based receptor. Therefore, the choice of which strategy to use can be done based on the hardware area and timing constraints of each architecture. The next step then consists in integrating the resultant model into a larger project in *Vivado^®^* Design Suite, a *Xilinx^®^* development environment for system-level integration and implementation [51], and then building it (place and routing and generating the bitstream). It is important to highlight that the maximum operating frequency, hardware area occupied, resources and power consumption are functions not only of the signal processing design but also of the target device.

In the present work, a *Xilinx^®^* Zynq UltraScale+ MPSoC (ZU15EG) is selected as the target device. The latter is mounted on a proprietary board that also has high speed analog to digital (ADC) and digital to analog (DAC) converters, blocks of DDR4 memories and a set of other peripherals not relevant to the present analysis. Within this context, despite being out of the scope of the present work to describe all the connections needed in the vhdl top level design, it is worth mentioning that the interface with the signal processing digital block is made via the AXI4 interface (defined and controlled by *ARM^®^*) which also contributes to the amount of resources needed. Table 1 lists the main resources consumed from the ZU15EG device. It is important to notice that the resources’ consumption presented also include the infrastructure demanded for the FPGA to communicate to the software inside the MPSoC. Unfortunately, due to the smaller hardware area of an older version of FPGA, the waveform generation designs could not be implemented in a Virtex 5 Integrated Circuit, and, as a consequence, could not be employed with the experimental radar PO1, described in the previous section.

Note that the Box–Muller approach consumes more hardware area, especially DSPs blocks, that are highly consumed by the receivers signal processing chain. Nevertheless, it is possible to say based on the results shown in Table 1 that the resources consumption is not a limiting factor to the generation of FM pseudo-random waveforms based on LFSR, evidencing its high feasibility for real-time Noise Radar applications.

Performance parameters such as the overall system’s latency and data throughput were respectively estimated as 62 clock cycles, $16\times f_{s}$ bits for all approaches, since the most responsible for that are the FIR filter and the DDS blocks, present in all architectures. With respect to timing constraints, the Worst Negative Slack of each design was 0.410 ns (Box–Muller), 0.250 ns (Sum, *n* = 2) and 0.450 ns (Sum, *n* = 4). Regarding the total on chip power, they all presented a similar 5.4 W power consumption and $30.3{\;}^{\circ }$C junction temperature.

## 4. Conclusions

This paper focused on investigating the performance of several random phase and frequency modulated waveforms, varying the stochastic properties of their modulating signals. An initial discussion over the basic properties of circular distributions that model the phase of a random vector with constant modulus was performed, highlighting the particularities with respect to Noise Radar applications. Widespread parameters such as Doppler tolerance, spectral efficiency, range resolution, mutual information rate, and stationary side lobes levels at the matched filter output were addressed in the analysis, considering random modulating signals characterized by Wrapped Normal distributions. Additionally, an untapped parameter was also introduced in the study, the concept of expected peak side lobe level (PSL) at the matched filter output.

Second order moment closed form expressions were derived, characterizing the expected value of the transmit signals ambiguity function. The latter revealed that the Doppler shift does not influence the stationary side lobe level (or side lobe level at infinity—ISL). This result is particularly interesting, since the main lobe peak power of the PM/FM Noise Radar ambiguity function decreases with the increase of the Doppler frequency, while the stationary side lobe level remains the same and independent of the time shift, i.e., spread in the entire range-Doppler spectrum.

With respect to the system’s range profile, the analysis evidenced that it can be separated in three well defined regions: the main lobe, the stationary side lobes and a transient region. It was shown that, as long as the resultant transmit signals present the same bandwidth, the derived expressions describing the mainlobe and ISL are valid despite of the stochastic properties of the modulating signal. With respect to the transient region, on the other hand, it was shown that it is dependent both on the properties of the modulating signal as well as on the modulation procedure (phase or frequency).

Transmit signals with similar autocorrelation function and Power Spectral Density (first order moments) present transient regions at the matched filter output with varied duration, function of the matched filter output variance (second order moment). Unlike the ISL, which can be estimated under a stationary assumption, depending on the modulation signal stochastic parameters, it is likely that the random peak side lobe (PSL) will occur in the aforementioned transient region, between a nearly zero variance region (main lobe) and a stationary variance region (ISL).

It was shown that frequency modulated signals with rectangular power spectral density presented the smaller transient region, indicating lower peak side lobe levels, while phase modulated signals with rectangular power spectral density, presented a higher and longer transient region, indicating higher peak side lobe levels. If not properly taken into consideration, PSLs can severely deteriorate a radar system performance. Therefore, frequency modulation is more suitable than phase modulation for generating constant modulus random waveforms and should provide a system with better performance.

Closed form expressions characterizing the transmit signal’s bandwidth and the system’s range resolution were also derived. It was shown that, despite serving as a good approximation, the peak to stationary side lobe level ratio is not simply given by the transmit signal’s time × bandwidth product. Moreover, it was highlighted that, depending on the properties of the modulating signals, there is a considerable difference on system’s performance when considering its 3 dB bandwidth or its 90% power bandwidth. The derived closed form expressions may not only serve as solid ground for more efficient optimization algorithms, but also support the proper specification of the system’s spectral confinement requirements. Finally, practical aspects of the analyzed PM/FM pseudo-random waveforms were addressed, such as real time implementation, degree of randomness, and the effects caused by hardware distortion.

Wrapped Normal random processes, as the ones analyzed in the present work, are good sources of Noise Radar modulating signals, since, as shown, they are relatively easy to generate and provide closed form expressions characterizing the signals throughout the receiver processing chain. Nevertheless, other type of circular distributions can serve this purpose as well, leading to systems with varied performances. Circular distributions characterization have already been subject of many studies in several engineering fields [29,30,31], as well as the statistics of Complex Signals in general [52,53,54], knowledge that can be inherited by the Noise Radar community to help design systems with enhanced performance. The feasibility as well as the suitability of employing samples of different distributions of the same Symmetric Wrapped α-Stable family as modulating signals are thus good alternatives for future works possibilities.

## Figures and Tables

**Table 1:** FPGA implementation—consumed resources.

Site Type	Frequency Modulation		
	Box–Muller	Sum, n=2	Sum, n=4
Flip-Flops	53,880	53,473	53,537
LUTs	39,015	36,817	36,870
Block RAM Tile	163	155.50	155.50
DSPs	31	24	24

## Data Availability

Not applicable.
